# PI3K-targeting strategy using alpelisib to enhance the antitumor effect of paclitaxel in human gastric cancer

**DOI:** 10.1038/s41598-020-68998-w

**Published:** 2020-07-23

**Authors:** Kui-Jin Kim, Ji-Won Kim, Ji Hea Sung, Koung Jin Suh, Ji Yun Lee, Se Hyun Kim, Jeong-Ok Lee, Jin Won Kim, Yu Jung Kim, Jee Hyun Kim, Soo-Mee Bang, Jong Seok Lee, Hark Kyun Kim, Keun-Wook Lee

**Affiliations:** 1 Biomedical Research Institute, Seoul National University Bundang Hospital, Seongnam, 13620 Republic of Korea; 2 Department of Internal Medicine, Seoul National University Bundang Hospital, Seoul National University College of Medicine, 82 Gumi-ro 173 Beon-gil Bundang-gu, Seongnam, 13620 Republic of Korea; 3 National Cancer Center Graduate School of Cancer Science and Policy, National Cancer Center, Goyang, 10408 Republic of Korea

**Keywords:** Cancer, Cancer, Molecular medicine

## Abstract

*PIK3CA* mutations are frequently observed in various human cancers including gastric cancer (GC). This study was conducted to investigate the anti-tumor effects of alpelisib, a PI3K p110α-specific inhibitor, using preclinical models of GC. In addition, the combined effects of alpelisib and paclitaxel on GC were evaluated. Among the SNU1, SNU16, SNU484, SNU601, SNU638, SNU668, AGS, and MKN1 GC cells, three *PIK3CA*-mutant cells were predominantly sensitive to alpelisib. Alpelisib monotherapy decreased AKT and S6K1 phosphorylation and induced G_0_/G_1_ phase arrest regardless of *PIK3CA* mutational status. The alpelisib and paclitaxel combination demonstrated synergistic anti-proliferative effects, preferentially on *PIK3CA*-mutant cells, resulting in increased DNA damage response and apoptosis. In addition, alpelisib and paclitaxel combination potentiated anti-migratory activity in *PIK3CA-*mutant cells. Alpelisib partially reversed epithelial–mesenchymal transition markers in *PIK3CA*-mutant cells. In a xenograft model of MKN1 cells, the alpelisib and paclitaxel combination significantly enhanced anti-tumor activity by decreasing Ki-67 expression and increasing apoptosis. Moreover, this combination tended to prolong the survival of tumor-bearing mice. Our data suggest promising anti-tumor efficacy of alpelisib alone or in combination with paclitaxel in *PIK3CA*-mutant GC cells.

## Introduction

Gastric cancer (GC) is the fifth most common cancer and the third leading cause of death worldwide^1^. In South Korea, GC is the second most common cancer and its incidence is significantly higher than that in Western countries^2^. A substantial proportion of patients with GC is diagnosed at the advanced or metastatic stage and has a poor prognosis. Recent development of targeted agents has significantly improved the clinical outcome of patients with various advanced solid tumors, especially breast cancer, lung cancer, and some hematological malignancies. In the meantime, numerous clinical trials have been conducted on GC. However, most trials were not successful except those utilizing trastuzumab, ramucirumab, and nivolumab^3–5^. Therefore, there is huge and urgent need to develop novel therapeutic strategies for the management of patients with advanced or metastatic GC, based on specific targets in the cancer cells. The Cancer Genome Atlas Research Network previously identified that *PIK3CA* is the third most frequently mutated gene, following *TP53* and *ARID1A* in GC^6^. *PIK3CA* mutations are present in approximately 9–13% of patients with non-hypermutated GC and 32% of patients with hypermutated GC.

Phosphatidylinositol 3-kinase (PI3K) signaling pathway is important in cancer cell proliferation and survival^7^. PI3K contains regulatory p85 and catalytic p110 subunits. *PIK3CA* encodes p110α, which is a PI3K catalytic subunit. Mutant *PIK3CA* is known to promote the proliferation and invasion of human cancer cells^8^. We reported that *PIK3CA* mutations are associated with increased tumor aggressiveness and AKT activation in patients with GC^9^. *PIK3CA* mutations are also known to confer resistance against HER2-targeted therapy in patients with HER2-positive breast cancer^10,11^. In addition, recent studies showed that targeting *PIK3CA* mutations can overcome hormone therapy resistance in patients with hormone receptor-positive breast cancer^12,13^. Therefore, *PIK3CA* mutations need to be investigated further as a potential therapeutic target in cancers. Based on these backgrounds, this preclinical study was conducted to investigate the anti-tumor effects and the mechanisms of alpelisib (BYL719), a PI3K p110α-specific inhibitor, using in vitro and in vivo GC models. In addition, the combined effects of alpelisib and paclitaxel, which is a commonly used drug for GC patients, were evaluated to explore whether this combination could enhance anti-tumor effects on GC.

## Results

### Alpelisib exhibits more potent anti-proliferative effects against PIK3CA-mutant gastric cancer cells than wild-type cells

**Figure 1:**
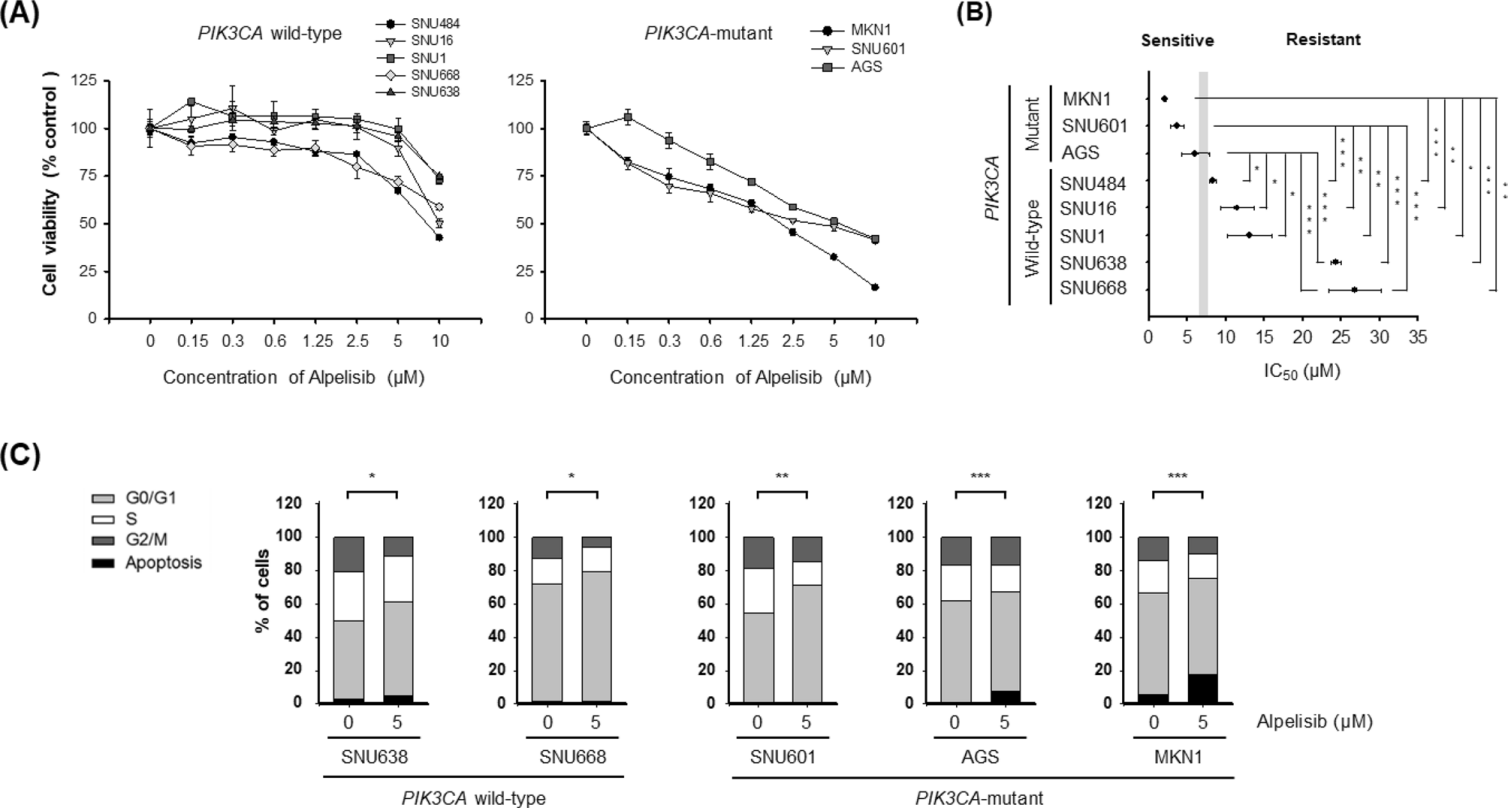
Effect of alpelisib on cell proliferation and cell cycle in gastric cancer cells. (**A**) Alpelisib at indicated concentrations was administered for 72 h to eight gastric cancer cell lines: SNU1, SNU16, SNU484, SNU601, SNU638, SNU668, AGS, and MKN1. All growth inhibition assays were repeated six times. (**B**) The IC_50_ values of each cell line were calculated using CalcuSyn software. The Student’s *t*-test was used to compare two independent groups. *p < 0.05; **p < 0.01; and ***p < 0.001. (**C**) Cell cycle analysis was conducted through flow cytometry after propidium iodide (PI) staining. Cells (1 × 10^6^) were seeded in 60-mm plates, and then treated with or without 5 μM of alpelisib for 24 h. Data are presented as histograms (bright gray, G_0_/G_1_ phase; white, S phase; dark gray, G_2_/M phase; black, sub-G_1_). The Student’s *t*-test was used to compare two independent groups. *p < 0.05; **p < 0.01; and ***p < 0.001.

We first summarized the mutational status of *PIK3CA* and other representative cancer-related genes in eight human GC cell lines (Supplementary Table 1) from Cancer Cell Line Encyclopedia (CCLE) database (https://portals.broadinstitute.org/ccle) and previous literature^14–17^. Among the GC cell lines, five were *PIK3CA* wild-type (SNU1, SNU16, SNU484, SNU638, and SNU668) and the other three were *PIK3CA*-mutant (SNU601, AGS, and MKN1). These cell lines were analyzed using CellTiter-Glo Luminescent Cell Viability Assay to quantify the anti-proliferative effects of alpelisib. Alpelisib treatment for 72 h inhibited cell proliferation in a dose dependent manner in both *PIK3CA* wild-type and mutant cells (Fig. 1A). Notably, the anti-proliferative effects of alpelisib were higher in *PIK3CA*-mutant cells (the half maximal inhibitory concentration [IC_50_] ranging from 2.1 to 5.2 μM) than *PIK3CA* wild-type cells (IC_50_ > 8.0 μM) (Fig. 1B and Supplementary Table 2).

### Alpelisib induces G_0_/G_1_ cell cycle arrest and apoptosis

To determine the effect of alpelisib on cell cycle, cell cycle analysis was performed in two *PIK3CA* wild-type (SNU638 and SNU668) and three *PIK3CA*-mutant (SNU601, AGS, and MKN1) cells (Fig. 1C). The flow cytometry data indicated that alpelisib treatment induced G_0_/G_1_ cell cycle arrest irrespective of *PIK3CA* mutational status. Notably, in *PIK3CA*-mutant cells (AGS and MKN1), sub-G_1_ fraction remarkably increased (*p* < 0.05), suggesting increased apoptosis by alpelisib in these cell lines.

### Alpelisib synergistically increases the anti-proliferative effects of paclitaxel in gastric cancer cells

**Figure 2:**
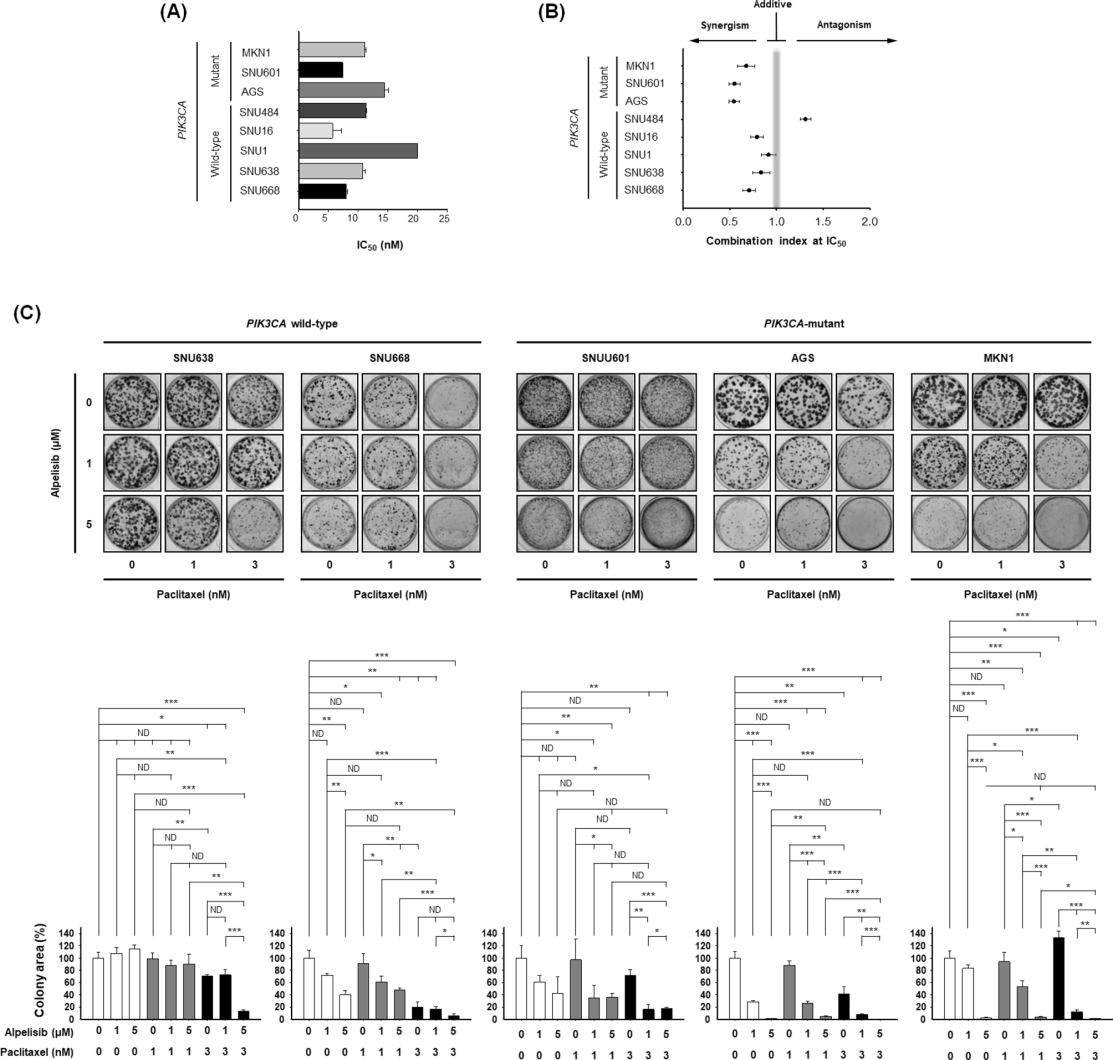
Combined effects of alpelisib and paclitaxel on cell proliferation and colony formation in vitro. (**A**) Five *PIK3CA* wild-type cells (SNU1, SNU16, SNU484, SNU638, and SNU668) and three *PIK3CA*-mutant cells (SNU601, AGS, and MKN1) were treated with paclitaxel (0, 0.125 nM, 0.25 nM, 0.5 nM, 1 nM, 2 nM, 4 nM, 8 nM, 10 nM, and 20 nM) for 72 h. The IC_50_ values were calculated using CalcuSyn software. Data expressed as mean ± standard deviation of three replicates. (**B**) The eight GC cell lines were exposed to increasing concentration of alpelisib and paclitaxel at a fixed ratio. The synergistic potential of alpelisib combined with paclitaxel was determined by calculating the combination index (CI) using CalcuSyn software according to Chou-Talalay method. The CI values < 1, = 1, and > 1 indicate synergistic, additive, and antagonistic effects, respectively. (**C**) Colony formation assays were conducted in two *PIK3CA* wild-type (SNU638 and SNU668) and three *PIK3CA*-mutant (SNU601, AGS, and MKN1) cells. The Student’s t-test was used to compare two independent groups. *p < 0.05; **p < 0.01; and ***p < 0.001.

We investigated whether alpelisib would potentiate the anti-tumor effects of paclitaxel in GC cells. First, we examined the anti-proliferative effects of paclitaxel in the eight GC cell lines (Fig. 2A). The IC_50_ values for MKN1, SNU601, AGS, SNU484, SNU16, SNU1, SNU638, and SNU668 cells were 11.1, 7.4, 14.4, 11.3, 5.7, 20.0, 10.8, and 7.9 μM, respectively. The sensitivity to paclitaxel differed among the cell lines and was associated with neither alpelisib sensitivity nor *PIK3CA* mutational status.

Further, the effect of paclitaxel and alpelisib combination on cell proliferation was evaluated using Chou-Talalay method^18^. The calculated combination index (CI) values were less than 1 in all the three *PIK3CA*-mutant cells (SNU601, AGS, and MKN1) and in four *PIK3CA* wild-type cells (SNU16, SNU1, SNU638, and SNU668), which indicated synergistic anti-proliferative effect of alpelisib and paclitaxel, especially in *PIK3CA*-mutant cells (Fig. 2B).

Next, colony formation assays were carried out to evaluate the anti-proliferative effects of alpelisib and/or paclitaxel on five GC cell lines (Fig. 2C): two *PIK3CA* wild-type (SNU638 and SNU668) and three *PIK3CA*-mutant cells (SNU601, AGS, and MKN1). The data for colony formation assay also reproduced the results of ATP-based cell viability assay: *PIK3CA*-mutant cells (SNU601, AGS, and MKN1) were highly sensitive to alpelisib, while alpelisib effect was relatively modest in *PIK3CA* wild-type cells (SNU638 and SNU668). In addition, alpelisib combined with paclitaxel significantly increased the anti-proliferative effect of paclitaxel in a dose dependent manner. Particularly, in *PIK3CA*-mutant cells (SNU601 and MKN1), 1 nM and 3 nM of paclitaxel monotherapy did not effectively inhibit colony formation. Nevertheless, the addition of alpelisib to paclitaxel treatment more potently suppressed colony formation in these two cell lines.

### Alpelisib and paclitaxel combination induces apoptosis in PIK3CA-mutant cells

**Figure 3:**
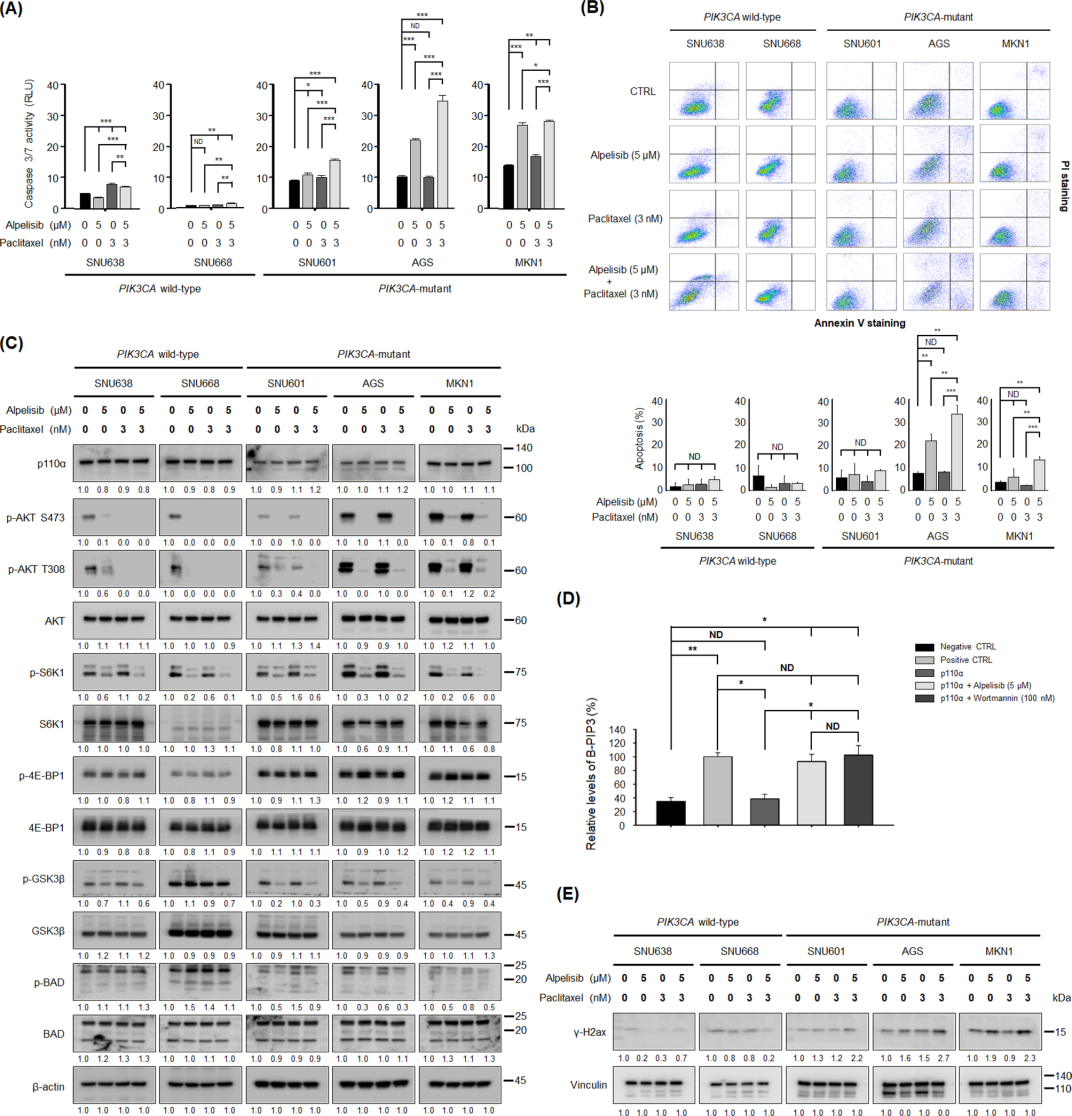
Combined effects of alpelisib and paclitaxel on caspase 3/7 activity, apoptosis, PI3K downstream molecules, PI3K p110α activity, and the expression levels of γ-H2ax in gastric cancer cells. (**A**) Caspase 3/7 activity (RLU, relative luminescence units) was quantified 24 h after alpelisib, paclitaxel, or their combination treatment in SNU638, SNU668, SNU601, AGS, and MKN1 cells. The Student’s *t*-test was used to compare two independent groups. *p < 0.05; **p < 0.01; and ***p < 0.001. (**B**) Apoptosis was evaluated by flow cytometry of Annexin V-propidium iodide (PI) double-stained gastric cancer cells after treatment with alpelisib and/or paclitaxel for 24 h. The Y-axis represents the PI-labeled population, whereas the X-axis represents the Annexin V positive cells. The left lower quadrant (Annexin V-, PI-) indicates normal cells, whereas the right lower quadrant (Annexin V+, PI−) and the right upper quadrant (Annexin V+, PI+) are the early and late apoptotic cells, respectively. The Student’s *t*-test was used to compare two independent groups. **p < 0.01; and ***p < 0.001. (**C**) SNU638, SNU668, SNU601, AGS, and MKN1 cells were treated with 5 μM of alpelisib and/or 3 nM of paclitaxel for 30 min. Expression levels of PI3K p110α, p-AKT S473, p-AKT T308, AKT, p-S6K1, S6K1, p-4E-BP1, 4E-BP1, p-GSK3β, GSK3β, p-BAD, and BAD were determined by Western blot. β-actin was the control. Protein expression was analyzed by ImageJ software. (**D**) The biotinylated-PIP3 (B-PIP3) was set as 100%. The kinase reactions with or without alpelisib or wortmannin were referenced to the B-PIP3 signal to calculate the relative effects of PI3K inhibitors. The recombinant GRP-1 protein was used as the capture protein. GRP-1 bound to the glutathione-coated plate competitively captures either the PIP3 generated by the kinase reaction or the B-PIP3. The Student’s *t*-test was used to compare two independent groups. *p < 0.05 and **p < 0.01. (**E**) SNU638, SNU668, SNU601, AGS, and MKN1 cells were treated with 5 μM of alpelisib and/or 3 nM of paclitaxel for 72 h. The expression levels of γ-H2ax was determined by Western blot. Vinculin was the control. Protein expression was analyzed by ImageJ software.

The combined effect of alpelisib and paclitaxel on apoptosis was examined using caspase 3/7 assay. Caspase 3/7 activity significantly increased after alpelisib and paclitaxel combination treatment (Fig. 3A), especially in *PIK3CA*-mutant cells rather than in *PIK3CA* wild-type cells. These results were confirmed by Annexin V-propidium iodide (PI) double staining assay (Fig. 3B), showing a strong induction of apoptosis after 24 h of alpelisib and paclitaxel combination treatment compared to alpelisib or paclitaxel monotherapy groups in *PIK3CA*-mutant cells, but not in *PIK3CA* wild-type cells.

### Alpelisib abrogates the phosphorylation of PI3K downstream molecules

We tested whether alpelisib and/or paclitaxel altered the PI3K p110α expression levels in *PIK3CA-*mutant and wild-type GC cells. Our data showed that alpelisib alone, paclitaxel alone, and alpelisib plus paclitaxel combination had no effect on the PI3K p110α expression (Fig. 3C). Instead, alpelisib significantly decreased the ‘PI(3,4)P2 (PIP2) to PI(3,4,5)P3 (PIP3) conversion’ in the PI3K p110α activity assay (Fig. 3D), suggesting that 5 μM of alpelisib was sufficient to repress the PI3K p110α activity.

A previous study in *PIK3CA*-mutant breast cancer demonstrated that mTORC1 inhibition is required for sensitivity to PI3K p110a inhibitors^19^. S6K1 phosphorylation is a representative marker for mTORC1 activity. Therefore, to test the inhibitory activity of alpelisib and paclitaxel combination on PI3K and mTORC1 signaling, we analyzed the expression of PI3K and mTORC1 signaling proteins including AKT, S6K1, and 4E-BP1 by Western blot (Fig. 3C). Regardless of *PIK3CA* mutational status, alpelisib monotherapy decreased AKT and S6K1 phosphorylation. Moreover, in *PIK3CA*-mutant cells, GSK3β and BAD phosphorylation was potently abrogated by alpelisib alone; however, this was not evident in *PIK3CA* wild-type cells. In addition, neither alpelisib nor paclitaxel affected 4E-BP1 phosphorylation.

### Alpelisib and paclitaxel combination further increases DNA damage in PIK3CA-mutant gastric cancer cells

To quantify the DNA damage, we analyzed the phosphorylation of the histone protein H2ax (γ-H2ax) (Fig. 3E). The levels of γ-H2ax were not apparently increased by alpelisib and/or paclitaxel treatment in *PIK3CA* wild-type cells. In contrast, in *PIK3CA*-mutant cells, alpelisib alone or paclitaxel alone increased γ-H2ax levels with an exception of paclitaxel monotherapy in MKN1 cells. Moreover, the combination treatment of alpelisib and paclitaxel further increased γ-H2ax levels compared with alpelisib alone or paclitaxel alone in *PIK3CA*-mutant cells.

### Alpelisib and paclitaxel combination potentiates anti-migratory activity in gastric cancer cells via independent mechanisms

**Figure 4:**
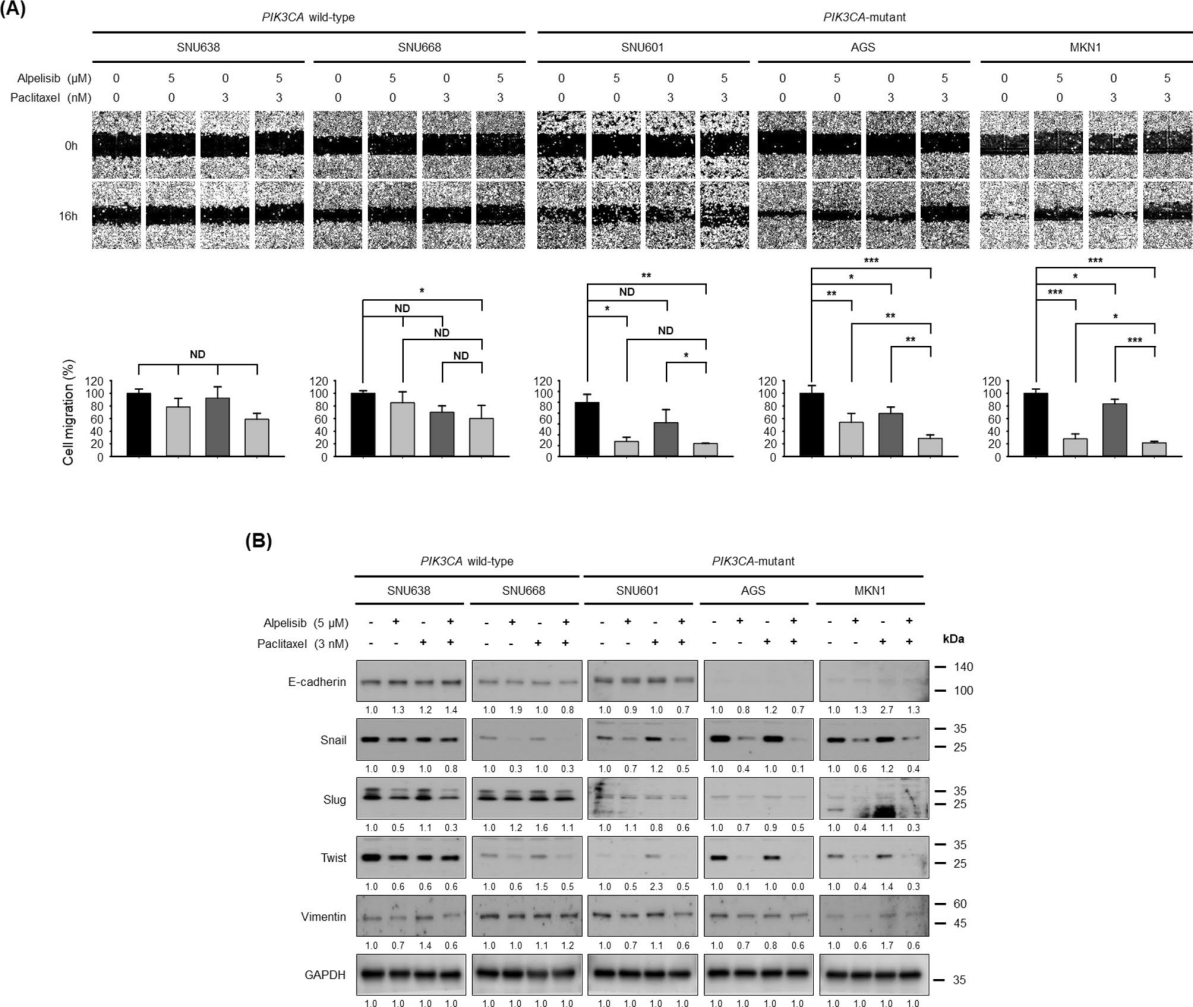
Combined effects of alpelisib and paclitaxel on cell migration and epithelial–mesenchymal transition markers expression. (**A**) The migration of SNU638, SNU668, SNU601, AGS, and MKN1 cells was assessed by the wound healing assay after 16 h of treatment. Representative images of the scratched areas are shown. Cell migration was quantified with ImageJ software. The Student’s *t*-test was used to compare two independent groups. *p < 0.05; **p < 0.01; and ***p < 0.001. (**B**) SNU638, SNU668, SNU601, AGS, and MKN1 cells were treated with alpelisib combined with paclitaxel for 16 h. Expression levels of E-cadherin, Snail, Slug, Twist, and vimentin were determined by Western blot. GAPDH was the control. Protein expression was analyzed by ImageJ software.

The effect of alpelisib and paclitaxel combination on migration was evaluated using wound healing assay (Fig. 4A). In *PIK3CA* wild-type cells, 5 μM of alpelisib alone did not significantly affect cell migration. However, in *PIK3CA*-mutant SNU601, AGS, and MKN1 cells, alpelisib alone significantly suppressed migration by 34.1%, 48.2%, and 60.9%, respectively. Additionally, in the five cell lines, 3 nM of paclitaxel alone showed only little influence on cell migration. Instead, alpelisib and paclitaxel combination resulted in potent anti-migratory activity in all three *PIK3CA-*mutant cells, while this was relatively less potent in *PIK3CA* wild-type cells.

To unveil the mechanisms potentiating the anti-migratory activity of alpelisib plus paclitaxel combination in GC cells, the epithelial to mesenchymal transition (EMT) markers including E-cadherin, Snail, Slug, Twist, and vimentin were evaluated by Western blot (Fig. 4B). Alpelisib inhibited the expression of mesenchymal markers Snail, Slug, Twist, and vimentin in most GC cell lines, while the expression of epithelial marker E-cadherin showed inconsistent changes by alpelisib for each cell line. In contrast, paclitaxel alone showed no clear and consistent effect on the expression of both epithelial and mesenchymal markers. When these EMT markers were evaluated by Western blot, we could not find significant or consistent EMT marker changes by alpelisib plus paclitaxel combination compared with alpelisib alone.

### Alpelisib and paclitaxel combination shows potent anti-tumor activity in vivo

In prior in vitro experimental data, we found that *PIK3CA*-mutant GC cells were preferentially sensitive to alpelisib compared to *PIK3CA* wild-type cells. Therefore, it seemed like the target population of alpelisib should be *PIK3CA*-mutant GC, rather than *PIK3CA* wild-type GC. Thus, we conducted xenograft experiments using *PIK3CA*-mutant MKN1 cells.

**Figure 5:**
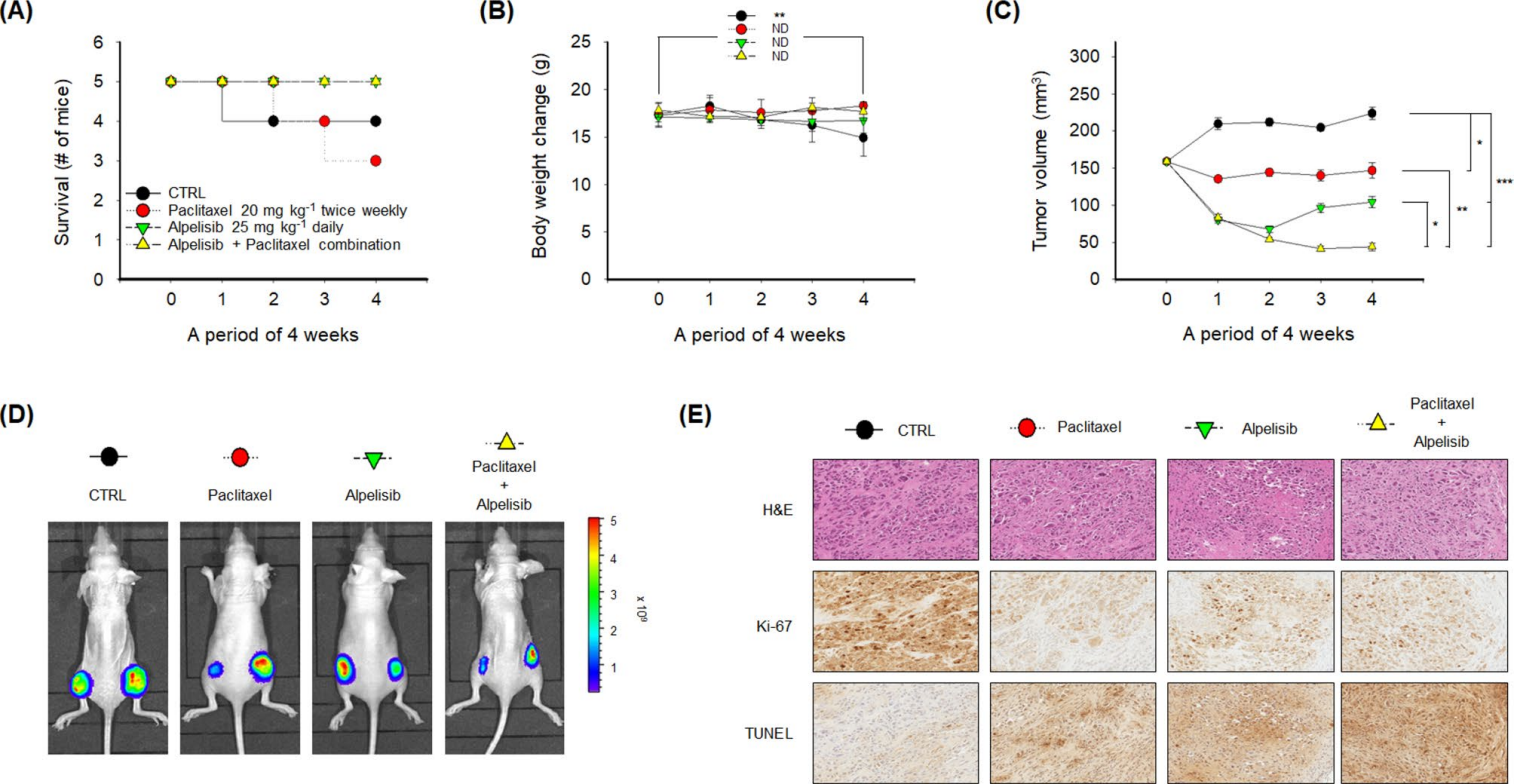
In vivo anti-tumor activity and tolerability of alpelisib and paclitaxel combination in the MKN1 xenograft model. (**A**) The survival of mice was analyzed by the Kaplan–Meier method. Five mice were assigned to each treatment group. (**B**) For 4 weeks of treatment period, the change in body weight was plotted according to the treatment groups. For the in vivo study, the values were presented as the mean ± standard error of the mean. The Student’s *t*-test was used to compare two independent groups. **p < 0.01. (**C**) Tumor volumes were measured every week for 4 weeks. The Student’s *t*-test was used to compare two independent groups. *p < 0.05; **p < 0.01; and ***p < 0.001. (**D**) The representative images of MKN1 luciferase tumor-bearing mice indicate the size of tumors at 5 weeks. d-luciferin was injected into mice for 10 min and was analyzed using in vivo optical imaging system 200 (IVIS 200). (**E**) Formalin-fixed paraffin-embedded tumor sections were stained with H&E, Ki-67, and terminal deoxynucleotidyl transferase dUTP nick end labeling (TUNEL) antibodies.

Using a mouse xenograft model of *PIK3CA*-mutant MKN1 cells that stably express luciferase, the in vivo anti-tumor activity of alpelisib and paclitaxel combination was evaluated. Each of the 25 mice that had two tumors on both flanks was assigned to each treatment group: (A) control group (n = 5), (B) alpelisib monotherapy group (n = 5), (C) paclitaxel monotherapy group (n = 5), and (D) combination group (n = 5). During the 4-week treatment period, there was no death event in alpelisib and paclitaxel combination group (Fig. 5A), while there were one and two deaths in the control group and paclitaxel monotherapy group, respectively. Moreover, the body weight of the control group significantly decreased after 4 weeks compared to the other three treatment groups (Fig. 5B). There was no significant change in body weight among the other three treatment groups during the 4-week treatment period.

The volume of tumors in the control group increased during the follow-up (Fig. 5C, D). Alpelisib or paclitaxel monotherapy significantly retarded tumor growth compared to the control group (*p* < 0.05). The combination of alpelisib and paclitaxel more potently inhibited tumor growth compared to alpelisib or paclitaxel monotherapy with a statistical significance (*p* < 0.05).

Formalin-fixed paraffin-embedded tissues from MKN1 tumors were stained with hematoxylin and eosin (H&E) for assessment of tumor areas. Ki-67 expression apparently decreased in the three treatment groups compared to the control group. In alpelisib or paclitaxel monotherapy group, terminal deoxynucleotidyl transferase dUTP nick end labeling (TUNEL) expression increased compared to the control group. Moreover, alpelisib and paclitaxel combination treatment apparently increased TUNEL expression compared to alpelisib or paclitaxel monotherapy group (Fig. 5E).

## Discussion

The aim of our study was to elucidate the therapeutic implication of *PIK3CA* mutations with PI3K p110α-specific inhibitor alpelisib combined with paclitaxel in GC. Here, we showed that *PIK3CA*-mutant SNU601, AGS, and MKN1 GC cells were predominantly sensitive to alpelisib. The molecular function of alpelisib was shown to inhibit the activity of PI3K p110α by binding to the ATP-binding pocket domain, resulting in inhibition of ‘PIP2 to PIP3 conversion’ (Fig. 3C, D), rather than direct inhibition of PI3K p110α expression levels^20^. Alpelisib monotherapy decreased AKT and S6K1 phosphorylation and induced G_0_/G_1_ cell cycle arrest notwithstanding *PIK3CA* mutational status. Moreover, in *PIK3CA*-mutant GC cells, GSK3β and BAD phosphorylation was potently abrogated by alpelisib alone, which was not evident in *PIK3CA* wild-type cells. Alpelisib in combination with paclitaxel demonstrated synergistic anti-proliferative effects, preferentially in *PIK3CA*-mutant GC cells, resulting in increased DNA damage response and apoptosis. In mouse xenograft model of *PIK3CA*-mutant MKN1 GC cells, alpelisib combined with paclitaxel significantly enhanced anti-tumor activity by decreasing Ki-67 expression and increasing TUNEL expression. Moreover, this combination prolonged the survival of tumor-bearing mice during 4 weeks of treatment period without resulting in significant change in body weight.

To explain the mechanism of the synergism, we hypothesized that DNA damage of the GC cells would be potentiated by the alpelisib and paclitaxel combination. A previous study showed that alpelisib leads to an increased nucleotide depletion-mediated DNA damage of cancer cells and subsequently induces cancer cell death^21^. In addition, it is very well known that paclitaxel inhibits the progression of mitosis and that prolonged mitotic checkpoint arrest causes the repression of DNA synthesis and apoptosis^22^. As shown in Fig. 3E, the levels of γ-H2ax were further increased by the combination treatment in *PIK3CA*-mutant GC cells, compared with alpelisib or paclitaxel alone. However, the increase was not apparent in *PIK3CA* wild-type GC cells. These data suggest that the increased DNA damage response could be a mechanism of synergistic anti-tumor effect of the combination.

To unveil the mechanisms involved in the potentiation of anti-migratory effect of alpelisib and paclitaxel combination, we conducted additional experiments to evaluate the expression of EMT-associated proteins including E-cadherin, Snail, Slug, Twist, and vimentin (Fig. 4B). Alpelisib partially reversed EMT phenotype in *PIK3CA*-mutant GC cells. However, paclitaxel alone did not have consistent influence on the expression of both epithelial and mesenchymal markers. Moreover, there was no evidence of synergistic changes in EMT marker expression by the alpelisib and paclitaxel combination. A previous study demonstrated that paclitaxel inhibits cell motility via direct microtubule inhibition^23^. Therefore, it is speculated that the anti-migratory mechanism of alpelisib may be different from that of paclitaxel. Nevertheless, the anti-migratory effects were potentiated in our experiments when both alpelisib and paclitaxel were combined (Fig. 4A).

*PIK3CA* mutations are frequently observed in various cancers including breast, colorectal, endometrial, and head and neck cancers as well as GC^24^. Recent development of PI3K inhibitors has enlightened the possibility of targeted therapy in patients harboring *PIK3CA* mutations^25^. However, the clinical development of pan-PI3K and PI3K-mTOR dual inhibitors has not been successful, possibly due to significant dose-limiting toxicities and modest anti-tumor activities^26–28^. In contrast, PI3K isoform p110α-specific inhibitors such as alpelisib and INK1402 exhibited higher activity in *PIK3CA*-mutant tumors with decreased off-target toxicities^29–31^. Recently, SOLAR-1 study demonstrated that the combination of alpelisib and fulvestrant, a selective estrogen receptor degrader, prolonged progression-free survival among patients with *PIK3CA*-mutated, hormone receptor-positive, HER2-negative advanced breast cancer^13^. Based on these data, alpelisib combined with other cytotoxic or targeted agents could be a good strategy in managing patients with *PIK3CA*-mutant GC.

Our group previously reported that *PIK3CA* mutations were associated with increased tumor aggressiveness and AKT activation in patients with GC^9^. Therefore, alpelisib could be a potential therapeutic option for these patients. In GC, previous phase II studies of everolimus, an mTOR inhibitor, showed promising results^32,33^. However, subsequent phase III GRANITE-1 trial failed to demonstrate the superiority of everolimus over placebo^34^. The GRANITE-1 study included all comers without selection using specific biomarkers including *PIK3CA* mutation or amplification, *PTEN* loss, and other genetic alterations that lead to mTOR activation. The failure of GRANITE-1 study might have been attributed to lack of biomarker selection in patients with GC. Therefore, we suggest that future clinical trials targeting PI3K signaling pathway of GC should enroll patients based on specific biomarkers.

Paclitaxel is one of the standard cytotoxic agents for the treatment of patients with metastatic or recurrent GC^4^. There has not been any study evaluating the efficacy of PI3K inhibitor combined with paclitaxel in patients with *PIK3CA*-mutant GC. However, in patients with head and neck cancer, buparlisib (BKM120), pan-PI3K inhibitor combined with paclitaxel demonstrated promising clinical efficacy and manageable safety profiles in phase III BERIL-1 study^35^. In the colony formation assays of our study (Fig. 2C), paclitaxel monotherapy was not effective in SNU601 and MKN1 GC cells. However, in combination with alpelisib, the efficacy of paclitaxel significantly improved. Therefore, the alpelisib and paclitaxel combination could be a rational strategy in *PIK3CA*-mutant GC, as shown in this preclinical study.

Interestingly, even in *PIK3CA* wild-type GC cells, alpelisib could abrogate the phosphorylation of AKT and S6K1. However, the abrogation of GSK3β and BAD phosphorylation by alpelisib was preferentially observed in *PIK3CA*-mutant cells, not in *PIK3CA* wild-type cells. The difference in GSK3β and BAD phosphorylation may explain the difference in the sensitivity to alpelisib between *PIK3CA*-mutant and wild-type GC cells. In line with this finding, alpelisib-induced apoptosis was more prominent in *PIK3CA*-mutant cells than in *PIK3CA* wild-type cells, which was potentiated by adding paclitaxel. Because paclitaxel is a tubulin inhibitor during mitosis, it is rational that paclitaxel would not influence the phosphorylation of PI3K signaling pathway molecules.

In conclusion, alpelisib alone or in combination with paclitaxel demonstrated promising anti-tumor activity in in vitro and in vivo models of *PIK3CA*-mutant GC via inactivating PI3K down-stream molecules, increasing DNA damage response, and apoptosis. In addition, anti-migratory effects were potentiated with the combination of alpelisib and paclitaxel in GC cells. Our data suggest that this novel combination warrants further clinical investigations in patients with *PIK3CA*-mutant GC.

## Methods

### Human cell lines and reagents

Five GC cell lines, SNU1, SNU16, SNU484, SNU638, and MKN1 were purchased from the Korean Cell Line Bank (KCLB; Seoul, Republic of Korea). The other three cell lines SNU601, SNU668, and AGS were kindly provided by Prof. Yung-Jue Bang at Seoul National University, Seoul, Republic of Korea. The most recent authentication of each cell line was performed using ‘AmpFLSTR Identifiler PCR Amplification Kit (Catalog no. 4322288; Applied Biosystems, Foster, CA, USA)’ by the KCLB on Nov. 22, 2016. The 3530xL DNA Analyzer (Applied Biosystems) and the GeneMapper v5.0 software (Applied Biosystems) were used for DNA fingerprinting analysis. Alpelisib was provided by Novartis Pharma AG (Basal, Switzerland). Paclitaxel was purchased from Selleckchem (Houston, TX, USA). Roswell Park Memorial Institute (RPMI)-1640, phosphate buffered saline (PBS), fetal bovine serum (FBS), penicillin–streptomycin (P/S), and trypsin–EDTA were purchased from Gibco (Gaithersburg, MD, USA). Epidermal growth factor (EGF), fibroblast growth factor 2 (FGF2), mitomycin C (MMC), PI, RNase, and isopropanol were purchased from Sigma-Aldrich (St Louis, MO, USA). Antibodies specific for p-AKT S473, p-AKT T308, AKT, p-S6K1, p-4E-BP1, 4E-BP1, E-cadherin, Snail, Slug, vimentin, vinculin, γ-H2ax, and β-actin were purchased from Cell Signaling Technology (Danvers, MA, USA). PI3K p100α, S6K1, p-GSK3β, GSK3β, p-BAD, BAD, Twist, and GAPDH antibodies were purchased from Santa Cruz Biotechnology (Santa Cruz, CA, USA).

### Cell viability assays, IC_50_ calculation, and combination studies

Eight human GC cell lines were incubated overnight in 96-well plates containing RPMI-1640 and 10% FBS, at a density of 5 × 10^3^ cells per well. Cells were treated with alpelisib and/or paclitaxel for 72 h. The cell viability was detected using the CellTiter-Glo Luminescent Cell Viability Assay (Promega, Madison, WI, USA). CellTiter-Glo reagent was added to the 96-well plate and incubated for 3 h at 37 °C. Next, luminescence was measured with SpectraMax L microplate reader (Molecular Devices, Sunnyvale, CA, USA). CalcuSyn software was used to calculate the IC_50_ and the CI values (Biosoft, Ferguson, MO, USA). The CI values were determined based on dose–effect levels of median-effect plot of paclitaxel alone, alpelisib alone, and their combination at fixed molar concentration ratio. The CI values of < 1, 1, and > 1 indicate synergism, an additive effect, and antagonism, respectively.

### Analysis of cell cycle using flow cytometer

The five GC cell lines, SNU601, SNU638, SNU668, AGS, and MKN1, were plated in 100 mm^2^ culture dishes at a density of 1 × 10^6^ cells per dish for 48 h, and then treated with or without 5 μM of alpelisib. Next, the cells were harvested at 24 h by trypsinization, centrifugation, and fixation with 70% ethanol. A staining solution containing 0.05 μg/mL of PI and 0.2 mg/mL of RNase was used for DNA staining. BD FACSCalibur (Franklin Lakes, NJ, USA) was used for cell cycle analysis. The flow cytometry data was processed using BD CELLQuest software (Franklin Lakes).

### Colony forming assay

The five GC cell lines were seeded in 6-well culture plates containing RPMI-1640 supplemented with 10% FBS for 3 days at a density of 6 × 10^3^ cells (SNU601), 3 × 10^3^ cells (SNU638, SNU668, and MKN1), or 1 × 10^3^ cells (AGS) per well. Next, they were incubated with RPMI-1640 supplemented with 10% FBS and alpelisib at 37 °C with 5% CO_2_ for 21 days (SNU601, SNU638, SNU668, and MKN1) or 14 days (AGS). Growth media were changed every two days. The colonies of cells were stained with Coomassie brilliant blue for 30 min and washed with pre-cooled PBS. Colonies were examined using ChemiDoc Touch Imaging System (Bio-Rad, Hercules, CA, USA) and calculated using ImageJ software (NIH, Baltimore, MD, USA).

### Caspase 3/7 activity assay

The five GC cell lines were seeded in 96-well plates and treated with vehicle, alpelisib, and/or paclitaxel for 24 h at a density of 5 × 10^3^ cells per well. Caspase 3/7 activity was measured using Caspase-Glo 3/7 Assay (Promega) according to the manufacturer’s instructions. Luminescence was measured using a GloMax 96 Microplate Luminometer (Promega). Caspase 3/7 activities after alpelisib and/or paclitaxel treatment were shown as relative values to those after vehicle treatment.

### Western blot analysis

The protein samples were separated through sodium dodecyl sulphate–polyacrylamide gel electrophoresis (SDS-PAGE) and transferred onto polyvinylidene difluoride (PVDF) membrane (Millipore, Bedford, MA, USA)^36^. The membranes were blocked with 5% skim milk in Tris-buffered saline and 0.1% Tween-20 (TBST) for 1 h and incubated overnight with primary antibodies (1:2000) diluted in 1% bovine serum albumin (BSA) in TBST at 4 °C. After three washes with TBST, secondary antibodies that were conjugated with horseradish peroxidase were diluted (1:5000) in TBST containing 5% skim milk and incubated for 1 h at room temperature. The bands were visualized using ChemiDoc Touch Imaging System (Bio-Rad).

### Cell migration assay

The cells were seeded in 96-well plates at a density of 4 × 10^5^ cells per well and were allowed to proliferate for 24 h. Confluent monolayers were gently scratched using a WoundMaker (Essen BioScience, Ann Arbor, MI, USA). Cells at the partially detached edges of the scratch were allowed to reattach for additional 1 h. The cells were treated with 10 ng/mL of EGF, 10 ng/mL of FGF2, and 25 ng/mL of MMC. Next, microscope images were taken and defined as initial point of cell migration (T = 0 h). The cells were allowed to migrate for 16 h and the resulting migrated cells were analyzed using IncuCyte ZOOM (Essen BioScience) and quantified with ImageJ software.

### PI3K p110α activity assay

The specific activity of PI3K p110α was measured using a PI3Kinase Activity/Inhibitor ELISA Assay Kit (Millipore) according to the manufacturer’s instructions. The biotinylated-PIP3 (B-PIP3) was set as 100%. The kinase reactions with or without alpelisib or wortmannin (the general class I PI3K inhibitor) were referenced to the B-PIP3 signal to calculate the relative effects of PI3K inhibitors. The inhibitory effect on the p110α activity with wortmannin was compared to that with alpelisib. The PH domain of the general receptor of phosphoinositides 1 (GRP-1) protein binds to PIP3 with high affinity and specificity^37^. The recombinant GRP-1 protein was used as the capture protein. GRP-1 bound to the glutathione-coated plate competitively captures either the PIP3 generated by the kinase reaction or the B-PIP3. The captured B-PIP3 was detected after streptavidin-HRP conjugation using a spectrophotometer (Synergy H1, Bio Tek).

### Lentiviral vector transfection and selection of stable cells

The day before transfection, 2.5 × 10^6^ of 293T cells were seeded in 150 mm culture plates. Transfection was done with Lipofectamine 2000 according to the manufacturer's instructions using 2 μg of pLenti CMV/TO V5-LUC Puro (Addgene no. 19785), 7.5 μg of pMDLg/pRRE (12,251), 7.5 μg of pRSV/REV (12,253), and 5 μg of pMD2.G (12,259). The DNA:PEI complex (1:4) was incubated overnight with the cells in a final volume of 10 mL of Opti-MEM (Thermo Fisher Scientific, Waltham, MA, USA). The viral supernatants were harvested 48 h after transfection, filtered through 0.22 µm pore size filter, and stored at − 70 °C until use. For MKN1 cells infected with pLenti CMV/TO V5-LUC Puro vector, puromycin was added gradually to select stable cells for 2 weeks.

### Xenografts of human gastric cancer cells in athymic nude mice

All Balb/c athymic nude mice were housed in a specific pathogen-free facility at Seoul National University Bundang Hospital (SNUBH). All mouse experiments were approved by the Institutional Animal Care and Use Committee (IACUC) of SNUBH and performed in accordance with relevant guidelines and regulations. The project was approved by IACUC of SNUBH (no. 54-2018-023). For xenograft mouse studies, female athymic nude mice, weighing 26–28 g (5 weeks old), were purchased from Orient Bio Co. (Gapyeong, Republic of Korea). Mice were fed with NIH-07 rodent chow (Zeigler Brothers, Gardners, PA, USA). Animals were acclimated to temperature (22–25 °C), humidity (44.5–51.8%), and 12 h light/dark cycle for 1 week prior to use^38^. In vivo experiments were conducted using a mouse xenograft model of MKN1 cells that stably express luciferase. Cells at a dose of 1 × 10^7^ were implanted with Matrigel (BD Biosciences, San Jose, CA, USA) subcutaneously into both flanks of each mouse. The tumor volume was calculated using the following formula: (width^2^ × height)/2. When the tumor volume reached 150 to 200 mm^3^, the mice were randomly assigned to receive one of the following treatments: (A) daily oral administration of vehicle i.e., sterile water (control group), (B) daily oral administration of alpelisib (25 mg/kg/day) in sterile saline (alpelisib monotherapy group), (C) twice weekly peritoneal injection of paclitaxel (20 mg/kg/day) in sterile water (paclitaxel monotherapy group), and (D) alpelisib and paclitaxel combination (combination group). The mice were weighed, and tumor areas were measured throughout the study. Treatments continued for 4 weeks, and the mice were euthanized using CO_2_, weighed, and subjected to necropsy. The volume and weights of xenograft tumors were recorded. The selected tumor tissues were further examined through routine H&E staining and immunohistochemical (IHC) analysis^39^.

### In vivo optical imaging

Optical imaging was performed with a Xenogen IVIS 200 small animal imaging system (Alameda, CA, USA) and were analyzed using Living Image software 4.3.1 (Caliper Life Sciences), as previously described^40,41^. Anesthesia (2.5% isoflurane) was administered in an induction chamber with 100% oxygen at a flow rate of 1 L/min and maintained in Xenogen IVIS 200 with 1.5% mixture at 0.5 L/min. The athymic nude mice were injected with d-luciferin (100 mg/kg) dissolved in PBS (15 mg/mL) via intraperitoneal route. Subsequently, the mice were placed in prone position in the Xenogen IVIS 200 and 1 to 5 min frames were consecutively acquired until the maximum signal was reached (Fig. 5D).

### Immunohistochemistry

Paraffin-embedded tissue blocks from xenograft tumors were then extracted and cut to give representative sections of the tumors. Tumor tissue sections, mounted on poly-L-lysine-coated slide, were deparaffinized using standard methods. Endogenous peroxidase was blocked using 3% hydrogen peroxide in PBS for 10 min. Antigen retrieval was performed for 5 min in 10 mM sodium citrate buffer (pH 6.0) and heated at 95 °C in a steamer followed by cooling for 15 min, as previously described^39^. The slides were washed with PBS and incubated for 1 h at room temperature with a protein blocking solution (Vectastain ABC Kit, Vector Laboratories, Burlingame, CA, USA). Excess blocking solution was drained, and the samples were incubated overnight at 4 °C with one of the following: 1:500 dilution of Ki-67 antibody or 1:200 dilution of TUNEL antibody. The color was developed by exposing the peroxidase to diaminobenzidine reagent (Vector Laboratories), which forms a brown reaction product. The sections were then counterstained with Gill's hematoxylin (Sigma-Aldrich) for 1 min. The brown staining identified the expression of Ki-67 and TUNEL.

### Statistical analysis

For the in vitro study, experimental values are presented as the mean ± standard deviation. For the in vivo study, the values were presented as the mean ± standard error of the mean. The Student’s *t*-test was used to compare two independent groups. *p < 0.05; **p < 0.01; or ***p < 0.001.

## Supplementary information

Supplementary Information.
