# Supplementary material for: PI3K-targeting strategy using alpelisib to enhance the antitumor effect of paclitaxel in human gastric cancer
Source: Sci Rep. 2020 Jul 23;10:12308. doi: 10.1038/s41598-020-68998-w (PMC7378194; doi:10.1038/s41598-020-68998-w)

**Supplementary Information for**

**PI3K-targeting strategy using alpelisib to enhance the antitumor effect of paclitaxel in human gastric cancer**

Kui-Jin Kim^1,*^, Ji-Won Kim^2,*^, Ji Hea Sung^2^, Koung Jin Suh^2^, Ji Yun Lee^2^, Se Hyun Kim^2^, Jeong-Ok Lee^2^, Jin Won Kim^2^, Yu Jung Kim^2^, Jee Hyun Kim^2^, Soo-Mee Bang^2^, Jong Seok Lee^2^, Hark Kyun Kim,^3^ and Keun-Wook Lee^2,†^

^1^Biomedical Research Institute, Seoul National University Bundang Hospital, Seongnam 13620, Republic of Korea

^2^Department of Internal Medicine, Seoul National University Bundang Hospital, Seoul National University College of Medicine, Seongnam 13620, Republic of Korea

^3^National Cancer Center, National Cancer Center Graduate School of Cancer Science and Policy, Goyang 10408, Republic of Korea

^*^These authors contributed equally to this work.

**Running title:** Alpelisib combined with paclitaxel in GC

^†^**Corresponding author:** Keun-Wook Lee, MD, PhD

**Supplementary Table 1.** Mutational profiles in gastric cancer cells

|  | SNU1 | SNU16 | SNU484 | SNU601 | SNU638 | SNU668 | AGS | MKN1 |
| --- | --- | --- | --- | --- | --- | --- | --- | --- |
| *PIK3CA* | WT | WT | WT | E542K | WT | WT | E545A | E545K |
|  |  |  |  |  |  |  | E453K |  |
| *PTEN* | WT | WT | WT | WT | WT | WT | WT | WT |
| *EGFR* | WT | WT | WT | WT | WT | WT | WT | WT |
| *ERBB2* | WT | WT | WT | WT | WT | WT | WT | WT |
| *KRAS* | G12D | G12D | WT | G12D | WT | Q61K | G12D | WT |

Abbreviations: WT = wild-type

| **Group** | ***PIK3CA* status** | **Cell lines** | **IC_50_ (μM)** | ***p-*value  (MKN1 vs.)** | ***p*-value  (SNU601 vs.)** | ***p*-value  (AGS vs.)** |
| --- | --- | --- | --- | --- | --- | --- |
| 1 | Mutant | MKN1 | 2.042 | - | - | - |
|  |  |  | 2.189 |  |  |  |
|  |  |  | 2.078 |  |  |  |
| 2 | Mutant | SNU601 | 2.928 | - | - | - |
|  |  |  | 3.476 |  |  |  |
|  |  |  | 4.643 |  |  |  |
| 3 | Mutant | AGS | 3.939 | - | - | - |
|  |  |  | 6.765 |  |  |  |
|  |  |  | 7.282 |  |  |  |
| 4 | Wild-type | SNU484 | 8.491 | 0.000007 | 0.000599 | 0.049211 |
|  |  |  | 7.803 |  |  |  |
|  |  |  | 8.559 |  |  |  |
| 5 | Wild-type | SNU16 | 10.457 | 0.008999 | 0.002378 | 0.014443 |
|  |  |  | 14.054 |  |  |  |
|  |  |  | 10.000 |  |  |  |
| 6 | Wild-type | SNU1 | 12.939 | 0.011572 | 0.003062 | 0.011915 |
|  |  |  | 16.067 |  |  |  |
|  |  |  | 10.184 |  |  |  |
| 7 | Wild-type | SNU638 | 23.910 | 0.000101 | 0.000002 | 0.000038 |
|  |  |  | 25.000 |  |  |  |
|  |  |  | 24.000 |  |  |  |
| 8 | Wild-type | SNU668 | 25.989 | 0.003111 | 0.000168 | 0.000361 |
|  |  |  | 23.851 |  |  |  |
|  |  |  | 30.497 |  |  |  |

**Supplementary Table 2.** The statistical analysis of IC_50_ values between *PIK3CA*-mutant and wild-type cells.

**Supplementary Figure 1.** Uncropped Western blot images of indicated proteins in whole cell lysates from SNU638, SNU668, SNU601, AGS, and MKN1 cells treated with alpelisib and/or paclitaxel, related to Figure 3C and E


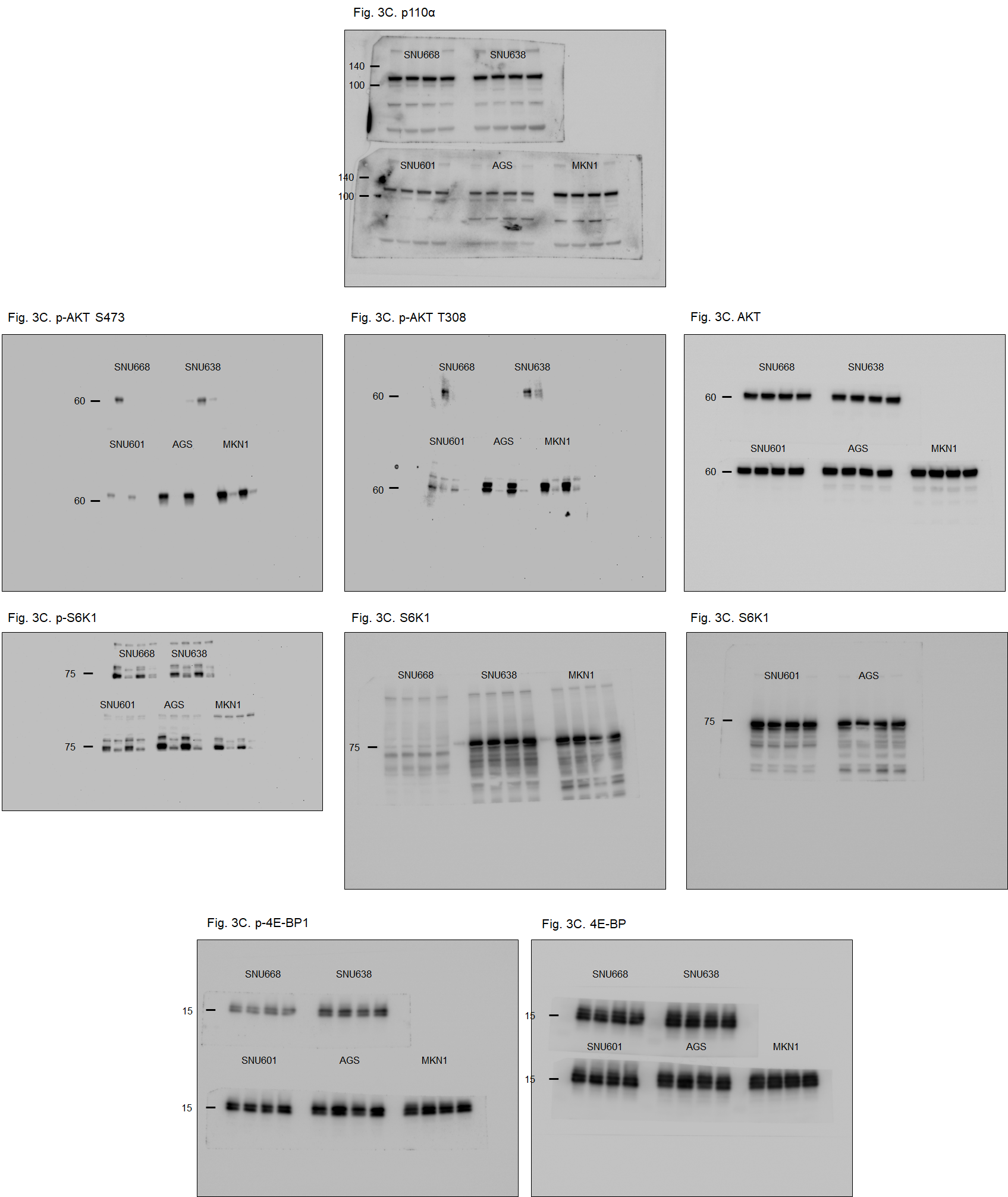

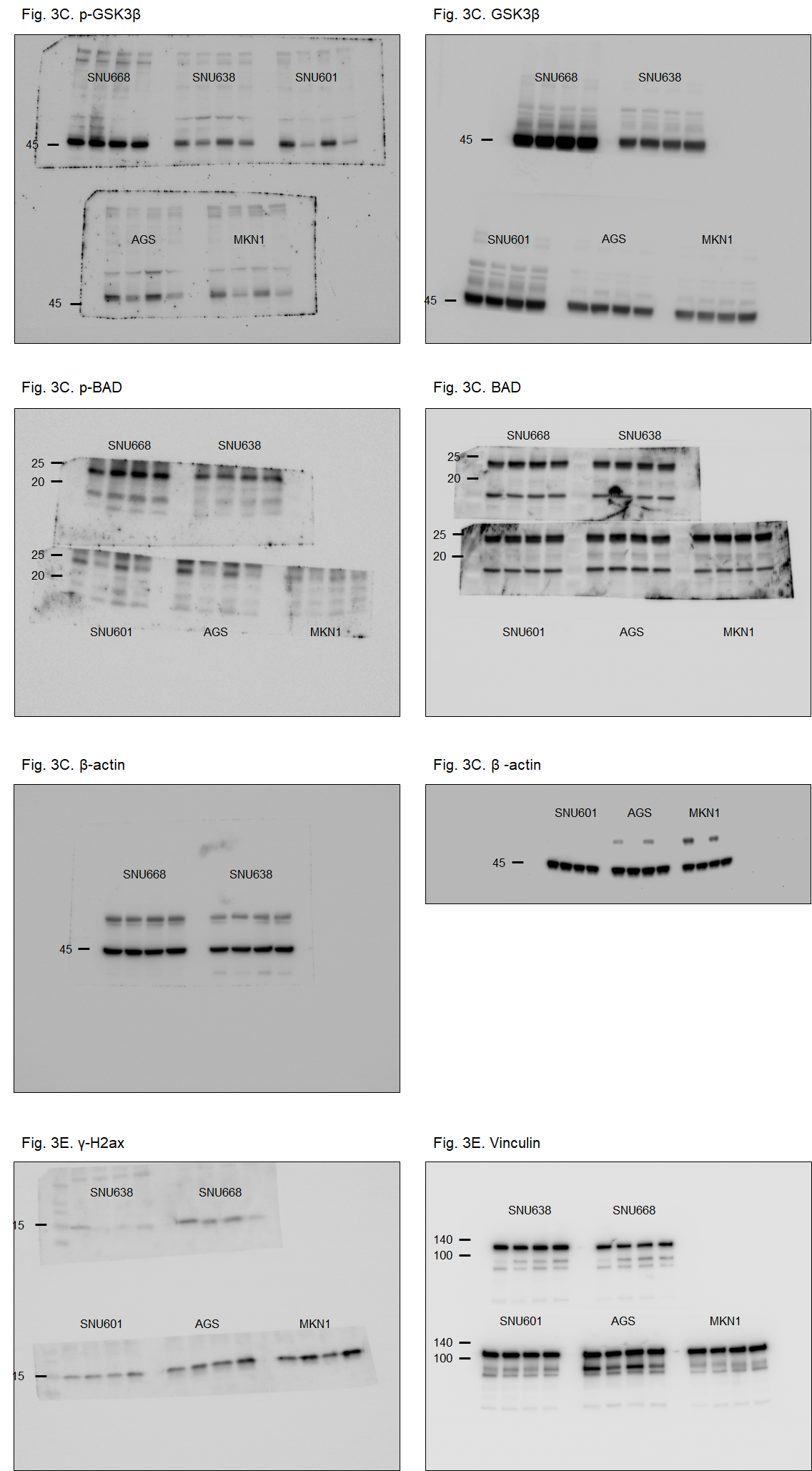


**Supplementary Figure 2.** Uncropped Western blot images of indicated proteins in whole cell lysates from SNU638, SNU668, SNU601, AGS, and MKN1 cells treated with alpelisib and/or paclitaxel, related to Figure 4B


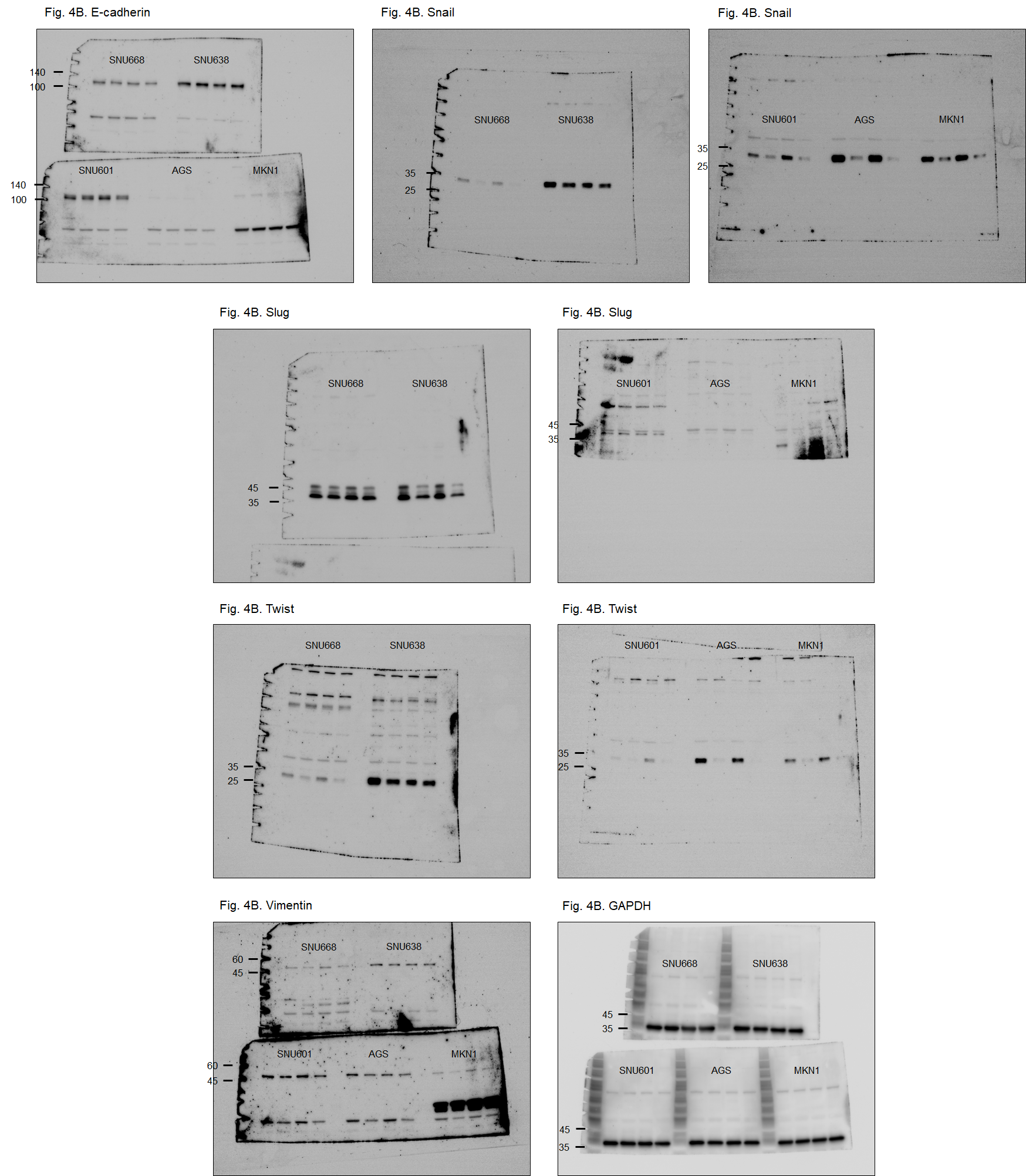

Supplement: Supplementary file 1 — Supplementary Information. [file 41598_2020_68998_MOESM1_ESM.docx]
